# Acute granulomatous interstitial nephritis in a patient with metastatic melanoma on targeted therapy with dabrafenib and trametinib*—*A case report

**DOI:** 10.1002/cnr2.1520

**Published:** 2021-08-05

**Authors:** Anna Krelle, Vinod Kalapurackal Mathai, Geoff Kirkland, Louise Nott, Matthew D. Jose, Karen Whale

**Affiliations:** ^1^ Department of Nephrology The Royal Hobart Hospital Hobart Tasmania Australia; ^2^ Department of Oncology The Royal Hobart Hospital Hobart Tasmania Australia; ^3^ School of Medicine The University of Tasmania Hobart Tasmania Australia; ^4^ Department of Pathology The Royal Hobart Hospital Hobart Tasmania Australia

**Keywords:** cancer management, melanoma, targeted therapy

## Abstract

**Background:**

Combination molecular targeted therapy with dabrafenib plus trametinib has been shown to improve progression‐free survival and overall survival in patients with BRAF V600 mutated unresectable or metastatic melanoma. In general, these agents are well tolerated. Kidney related adverse events are uncommon with only three case reports of acute interstitial nephritis and one case of a serious acute kidney injury. We report another case of interstitial nephritis related to these drugs.

**Case:**

A 37‐year‐old man diagnosed with metastatic melanoma (BRAF V600E mutation) who developed acute interstitial nephritis 5 years into his treatment with combination dabrafenib plus trametinib therapy. He presented with an asymptomatic acute kidney injury on routine surveillance pathology with a creatinine of 174 μmol/L (from baseline 80 μmol/L) and a corresponding estimated glomerular filtration rate (eGFR) of 42 ml/min/1.73 m^2^ (from a baseline >90 ml/min/1.73 m^2^) and microalbuminuria (albumin creatinine ratio [ACR] 8.5 mg/mmol). Renal biopsy revealed a granulomatous interstitial nephritis likely drug related. He was treated with prednisolone 1 mg/kg and ceased his targeted therapy with improvement in his renal function.

**Conclusion:**

Although rare, recognition of acute interstitial nephritis, a possible serious adverse outcome due to dabrafenib and trametinib is important and needs to be incorporated into current Australian cancer therapy guidelines.

## 1 BACKGROUND

Metastatic melanoma is associated with a poor prognosis with five‐year survival is less than 30%.^1^ BRAF activating mutations are present in approximately 40%–60% of patients with advanced melanoma.^2^ Dabrafenib is a genetically targeted small‐molecule inhibitor of oncogenic BRAF V600 mutation^3^ and trametinib is a potent, highly specific inhibitor of MEK1/MEK2, which is a downstream signalling partner of BRAF.^4^

The COMBI‐d trial, a phase III trial which randomised patients to either receive dabrafenib plus trametinib or dabrafenib plus placebo, found significant improvement in progression‐free survival (median 11.0 vs. 8.8 months, HR: 0.67 95% CI: 0.53–0.84) and overall survival (median 25.1 vs. 18.7 months, HR: 0.71, 95% CI: 0.55–0.92) with combination treatment as compared with dabrafenib alone.^5^ The COMBI‐v trial, another phase III trial, compared dabrafenib plus trametinib with vemurafenib alone on patients with untreated metastatic melanoma with a BRAF V600 mutation, which also showed significant benefit in 3‐year progression‐free survival (25% vs. 11%) for the combination treatment.^6^

The common early toxicities caused by anti BRAF agents include anorexia, fatigue, arthralgia and myalgia and mild alopecia.^7^ They may cause non‐infectious fever, less commonly associated with proliferative keratinocytic skin toxicities including squamous cell carcinoma, keratoacanthomas and plantar‐palmar hyperkeratosis and rarely associated with new primary non‐cutaneous malignancies such as colon and adenocarcinoma of the pancreas.^7^ The most serious adverse events reported for these agents are pulmonary and cardiac toxicity, which may manifest as arrhythmia, prolongation of QT interval, hypertension, cardiac ischemia and congestive cardiac failure.^7^ Some serious bleeding events have also been reported in case reports.^8^, ^9^

Few kidney side effects have been reported in relation to these therapies. Specifically, only three case reports of acute interstitial nephritis, and one case of a serious acute kidney injury (AKI)^10^, ^11^ in relation to this therapy exist. Australian cancer therapy guidelines currently do not report interstitial nephritis as a potential side effects of this combination therapy.

## 2 CASE

We present the case of a 37‐year‐old male was diagnosed with invasive malignant melanoma of his scalp in June 2011, with no evidence of metastatic disease and excision of his scalp lesion was undertaken (Breslow thickness 3.5 mm and Clark level 3). He worked in administration, had no other significant medical history at the time of his diagnosis. His baseline creatinine was 80 μmol/L with an estimated glomerular filtration (eGFR) rate of >90 ml/min/1.73 m^2^.

In 2012 he had recurrence in a left cervical lymph node and he underwent radical left neck dissection, with one out of twenty nodes showing metastatic melanoma but no evidence of extracapsular extension. He did not receive adjuvant radiotherapy. In 2014 he again developed recurrence of disease with PET scan in July 2014 showing left perineal subcutaneous nodule, which were resected, confirming metastatic melanoma and molecular analysis showed BRAF V600E mutation positivity. Following this resection, he was deemed disease free; however, early surveillance imaging several months later in November 2014 showed recurrence of the previous PET FDG avid metastases with metastatic disease involving bilateral axillary lymph nodes, supraclavicular nodes and anterior mediastinal nodes, multiple subcutaneous deposits, soft tissue deposit adjacent to the left hip joint, in addition to a large peritoneal deposit and some intramuscular deposits in the abdomen. Subsequently, in December 2014 he commenced targeted therapy with combination dabrafenib and trametinib.

The patient tolerated therapy well, with his only side effect being occasional non‐infectious febrile responses. First of these episodes started within first month of initiation of dabrafenib and trametinib, there were other two more episodes in first 3 months and a severe episode in November 2017 requiring hospital admission. These were successfully managed with brief cessation of his therapy, and short courses of corticosteroids and paracetamol. In October 2016 his FDG‐PET/CT demonstrated complete response and remained disease free since then. He showed no evidence of other organ dysfunction.

In late 2019, (5 years into his targeted therapy treatment) he had recurrence of a febrile illness which coincided with a FDG‐PET/CT demonstrating abnormal uptake in the neck (SUV max of left neck lesion 2.7). Biopsy was undertaken and showed evidence of necrotising granulomatous lymphadenitis with features possibly consistent with cat‐scratch lymphadenitis. Serology was performed for cat scratch, toxoplasmosis, CMV, which were all negative. QuantiFERON gold test was also negative. No other evidence of infection or recurrence of malignancy was identified, and he remained on his combination targeted therapy throughout this time.

**FIGURE 1:**
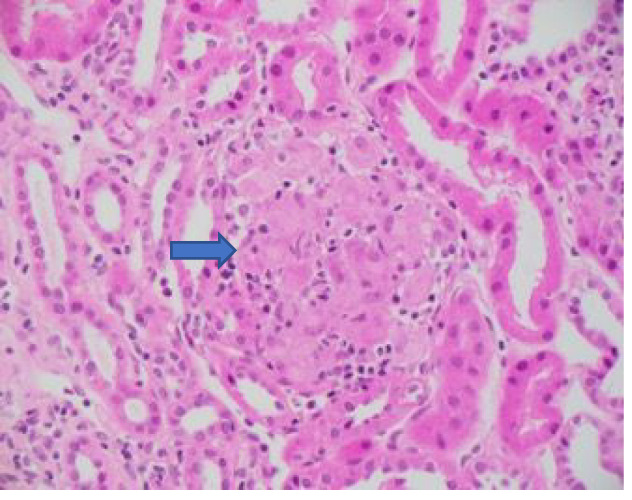
Non‐necrotizing granulomatous tubulointerstitial nephritis with small numbers of eosinophils involving cortex and medulla

However, several months later in March 2020, he was noted to have developed a significant AKI with a creatinine of 174 μmol/L and an eGFR of 42 ml/min/1.73 m^2^ (stage 2 as per KDIGO classification 2012)^12^ on routine surveillance biochemistry. He was mostly asymptomatic at that time. His urea was 10.4 mmol/L, bicarbonate 23 mmol/L and slight serum hypalbuminaemia at 34 g/dl. His liver function testing was unremarkable as were his other electrolytes. He was mildly anaemic with a haemoglobin of 124 g/L but normal white cell count and neutrophils. Of note, his serum eosinophils were normal. His urine microscopy and culture were unremarkable with a leucocyte count <3, erythrocytes of 8, with no growth. The urine ACR did however show evidence of microalbuminuria (8.5 mg/mmol). Other investigations for a cause of AKI were unrevealing, including a normal renal tract ultrasound without hydronephrosis. Over the next month there was no improvement in his kidney function and he subsequently underwent a kidney biopsy. The biopsy revealed a non‐necrotizing granulomatous tubulointerstitial nephritis with small numbers of eosinophils involving cortex and medulla (Figure 1). Three glomeruli showed fibrocellular crescents. Immunofluorescence was negative and there was no evidence of immune complexes on electron microscopy. In summary, the findings were consistent with granulomatous tubulointerstitial nephritis and a pauci‐immune glomerulonephritis with crescents; features in keeping with a drug reaction to BRAF/MEK inhibitors.

**FIGURE 2:**
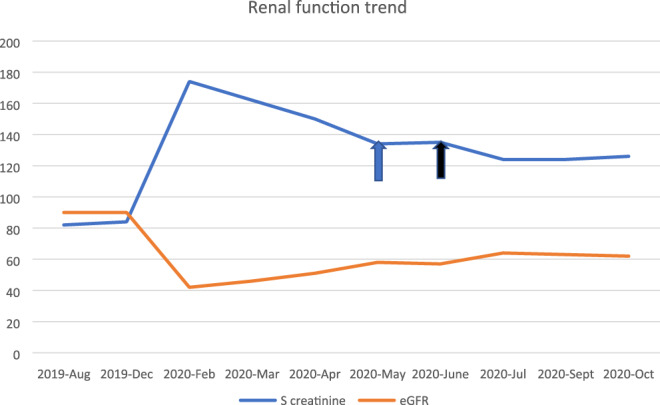
Trend in renal function (blue arrow—Prednisolone started, black arrow—dabrafenib and trametinib ceased)

In light of these biopsy findings an anti‐neutrophil cytoplasmic antibody (ANCA) was performed and returned negative. The cause of interstitial nephritis was thought secondary to his combination metastatic melanoma treatment BRAF and MEK inhibitors. He was initially continued on these agents while high dose prednisolone were commenced at 60 mg daily (1 mg/kg). Despite steroids, his kidney function did not show significant improvement and the decision was made to cease his targeted therapy while continuing steroid treatment. With cessation of his treatment, there has been improvement in his AKI, with a creatinine in July 2020 being 127 μmol/L and an eGFR 62 ml/min/1.73 m^2^ (Figure 2). He is currently undergoing a slow steroid weaning plan with careful monitoring. He remains off his anti BRAF/MEK treatment. He had a period of break from treatment following the AKI, his renal function improved but not quite to baseline. He then relapsed on PET scan which was biopsy proven and is being trialled on encorafenib and binimetinib with close observation, with a view that it may not be class effect. If there is further kidney function deterioration with these drugs, dual immunotherapy with ipilimumab and nivolumab will be the other option.

## 3 DISCUSSION

Our patient was commenced on dabrafenib and trametinib in December 2014, with normal baseline renal function, after he progressed with BRAF V600E mutated metastatic melanoma; he achieved excellent sustained disease control. His sudden renal impairment and microalbuminuria with biopsy proven granulomatous interstitial nephritis, after 5 years of this treatment is likely drug related can be attributed to this combination treatment as there were also no other possible culprit medications or other cause identified after careful investigations. This is supported by improvement in renal function after ceasing his targeted therapy. It is important to report this adverse reaction, given renal injury, in particular acute interstitial nephritis is not a well‐recognised side effect of this combination therapy.

Renal toxicity with dabrafenib was much less commonly reported compared with vemurafenib, another BRAF inhibitor,^7^ however there were reports of renal failure in <1% of patients treated with dabrafenib in the European summary of product characteristics of the drug.^13^ The majority of these cases were generally associated with fever and dehydration and responded well to dose interruption and supportive measures.^13^ There were reports of hyponatremia, hypophosphatemia, increased serum creatinine and hypokalemia with the dabrafenib and trametinib combination.^13^ The renal toxicities that can occur with BRAF inhibitor are allergic interstitial disease, acute tubular necrosis, proximal tubular damage (Fanconi's syndrome), hypophosphatemia, hyponatremia, subnephrotic range proteinuria, mild decrease in GFR (20%–40%), hematuria, proteinuria and white cell sediments.^13^

Jansen et al has reported a case with stage IIIB BRAF V600E mutant melanoma, treated with dabrafenib and trametinib who developed bilateral pedal oedema and erythematous back and upper arm rash, found to have AKI. The patient's skin and kidney biopsy indicated granulomatous inflammation and was attributed to the combination of dabrafenib and trametinib.^10^ Once combination treatment was ceased and steroids, patient had a good recovery of renal function.^10^ Ikesue et al reported a case of 89‐year‐old gentleman with baseline eGFR of 40 ml/min/1.73 m^2^ and recurrent BRAF V600E mutant melanoma, which had failed first line nivolumab treatment.^11^ This patient was started on dabrafenib and trametinib with reduction in their renal function eGFR to 29 ml/min/1.73 m^2^.^11^ They found that when the combination treatment was ceased, renal function returned to normal; it is not clear however if this is confounded by other factors.^11^ In our case, the AKI occurred several years later after initiation of dabrafenib and trametinib, unlike the other case reports. We believe this may be a rare side effect of these agents. It might be purely idiosyncratic drug reaction which can occur anytime or may be related to cumulative toxicity.

Management of acute interstitial nephritis due to targeted therapy is cessation of offending agents.^14^ In biopsy proven interstitial nephritis, steroid treatment^15^ and renal replacement therapy if required is the recommended approach.^14^ Mycophenolate can also be considered as a steroid sparing agent in this condition.^16^

## 4 CONCLUSION

This case highlights the importance of monitoring renal function when patients are on combination therapy of dabrafenib and trametinib. Our case of AKI secondary to interstitial nephritis occurred 5 years into the patient's treatment with molecular therapy, emphasising the ongoing need for surveillance throughout treatment courses, even if initially tolerated.

## CONFLICT OF INTEREST

The authors declare that they have no conflict of interest.

## AUTHOR CONTRIBUTIONS

All authors had full access to the data in the study and take responsibility for the integrity of the data and the accuracy of the data analysis. *Conceptualization*, G.K., L.N., M.D.J.; *Data Curation*, A.K., K.W.; *Investigation*, K.W.; *Resources*, V.K.M., L.N., K.W.; *Supervision*, G.K., L.N., M.D.J.; *Writing‐Original Draft*, A.K., V.K.M.; *Writing‐Review & Editing*, V.K.M.

## ETHICAL STATEMENT

Written consent has been obtained from the patient for the purposes of this case report. There are no objections from the institutions providing care for this patient in publishing this case report.

## Data Availability

The data that support the findings of this study are available on request from the corresponding author. The data are not publicly available due to privacy or ethical restrictions.
